# Give Thanks in All Circumstances? Gratitude Toward God and Health in Later Life After Major Life Stressors

**DOI:** 10.1093/geroni/igab046.1789

**Published:** 2021-12-17

**Authors:** Laura Upenieks, Joanne Ford-Robertson

**Affiliations:** 1 University of Texas at San Antonio, San Antonio, Texas, United States; 2 University of Texas at San Antonio, University of Texas at San Antonio, Texas, United States

## Abstract

Gratitude is foundational to well-being throughout the life course, and an emerging body of work suggests that older adults may be more inclined to attribute gratitude to a non-human target (God). Drawing on life course theory and Erikson’s lifespan development framework, we use data from a national sample of Christian older adults from the United States (N = 1,005) to examine whether gratitude towards God buffers the noxious health effects of the death of a loved one or personal illness. Results suggest that gratitude towards God tends to predict better age-comparative and global self-rated physical health in the aftermath of stress, a moderation effect which is partially mediated by stronger beliefs in God-mediated control (that God is a collaborative partner in dealing with problems). We conclude by proposing some interventions for clinicians and counselors centered around gratitude and religiosity that may assist older adults in coping with major life stressors.

